# The Averaged EMGs Recorded from the Arm Muscles During Bimanual “Rowing” Movements

**DOI:** 10.3389/fphys.2015.00349

**Published:** 2015-11-27

**Authors:** Tomasz Tomiak, Andriy V. Gorkovenko, Arkadii N. Tal'nov, Tetyana I. Abramovych, Viktor S. Mishchenko, Inna V. Vereshchaka, Alexander I. Kostyukov

**Affiliations:** ^1^Unit of the Theory of Sport and Motorics, Chair of Individual Sports, Gdansk University of Physical Education and SportGdańsk, Poland; ^2^Department of Movement Physiology, Bogomoletz Institute of Physiology, National Academy of SciencesKiev, Ukraine

**Keywords:** bimanual movements, two-joint movements, motor control, muscle synergy, electromyogram

## Abstract

The main purpose was to analyze quantitatively the the average surface EMGs of the muscles that function around the elbow and shoulder joints of both arms in bimanual “rowing” movements, which were produced under identical elastic loads applied to the levers (“oars”). The muscles of *PM group* (“*pulling” muscles*: elbow flexors, shoulder extensors) generated noticeable velocity-dependent dynamic EMG components during the pulling and returning phases of movement and supported a steady-state activity during the hold phase. The muscles of *RM group* (“*returning” muscles*: elbow extensors, shoulder flexors) co-contracted with *PM group* during the movement phases and decreased activity during the hold phase. The dynamic components of the EMGs strongly depended on the velocity factor in both muscle groups, whereas the side and load factors and combinations of various factors acted only in *PM group*. Various subjects demonstrated diverse patterns of activity redistribution among muscles. We assume that central commands to the same muscles in two arms may be essentially different during execution of similar movement programs. Extent of the diversity in the EMG patterns of such muscles may reflect the subject's skilling in motor performance; on the other hand, the diversity can be connected with redistribution of activity between synergic muscles, thus providing a mechanism directed against development of the muscle fatigue.

## Introduction

Simplified, constrained movements are often used to study the control of complex movement. (Gomi and Osu, 1998; Gribble and Ostry, 1998; Gribble et al., 2003; Tal'nov et al., 2014). To obtain the maximal extent of reproduction of the same movement paradigm, one of the most suitable procedures consists of the visual tracking of a basic movement parameter in accordance with a given command signal, which presents a desirable movement trajectory (Tal'nov et al., 1997, 1999, 2014; Gribble et al., 2003) or generated muscle force (Nijhof and Gabriel, 2006). The central commands directed to the muscles have typically been evaluated in experiments with many-fold repetitions of identical movement programs that were followed by an off-line averaging procedure applied to both the surface EMG activities recorded from the muscles that provide the movement and the basic mechanical parameters. Some motor control theories, such as the equilibrium state hypotheses (Feldman, 1966; Hogan, 1985; Feldman and Levin, 2009), are typically considered to be a single-valued correspondence between efferent activities directed to the joint muscles and its mechanical parameters. The analysis of the EMG patterns in the stereotyped isotorque single-joint movements simultaneously demonstrated evident movement-dependent uncertainties in the relationships between EMGs and the positioning parameters (Tal'nov and Kostyukov, 1994). The dynamics of the skeletal muscle behavior in the stretch reflex system are also essentially non-linear because they depend not only on the instantaneous values of neural activation and external load but also on the direction of previous movement and activation prehistory (Kostyukov, 1986, 1998; Herzog et al., 2006). Contractions of agonist and antagonist muscle groups evoke movements around a limb joint, whereas the muscle antagonists change their lengths in opposite directions during a movement. Because the dynamic muscle properties crucially depend on the direction of length change, the joint dynamics will reflect the complex interactions of the direction-dependent asymmetries in the behavior of the muscle antagonists.

The activation patterns of the antagonistic muscle groups are changed not only in different motor tasks but can markedly vary even in identical movements, depending on the balance between the activation intensities of the antagonists. It is quite clear that for a given movement amplitude, the required level of agonist activation will depend on the antagonist activity. Real movements quite often contain elements of co-activation; it is commonly accepted that the co-activation of antagonists increases the mechanical stiffness of the joint, which is especially important for the most proximal joints in multi-joint movements (Dounskaia et al., 2002). Increased stiffness is also important to overcome joint instability under different external loads; co-activation of antagonistic muscles is one of the main factors that improve movement precision (Gribble and Ostry, 1998; Gribble et al., 2003). The muscles from different joints can form different temporary groups that act in a synergic mode; some muscles participate in producing a given movement, whereas other muscles function in an opposing mode at various phases of the movement. It can be hypothesized that the patterns of the central commands that arrive at different muscles in the same synergic group will have both similarities and diversities. Two-joint movements present the simplest form of multi-joint movements that enable the comparison of dynamic and static EMG components in muscles that belong to different joints. Despite numerous studies devoted to the investigation of bimanual movements (Swinnen et al., 1996; Dounskaia et al., 1998, 2002; Soteropoulos and Perez, 2011; Gueugnon et al., 2014), we did not identify papers devoted to the quantitative comparative analysis of EMGs in identical muscles in both arms. The various problems regarding the hierarchical control of different coordination patterns in multi-joint movements are discussed in details elsewhere (Kelso, 1994; Dounskaia et al., 1998, 2002; Diedrichsen and Dowling, 2009).

The main aims of the current study include a detailed analysis of the EMG intensities of the muscles in the elbow and shoulder joints in both arms during the execution of identical bimanual pulling and returning movements of ramp-and-hold profile. We compared the EMG reactions in different muscles participating in these movements for two levels of external loading and three movement velocities. It was assumed that the extent of variability in EMG intensities would be different in different subjects even during the fulfillment of identical movements. Potential differences in the reactions of similar muscles in both arms during fulfillment of identical bimanual movements are considered.

## Methods

### Experimental setup

**Figure 1:**
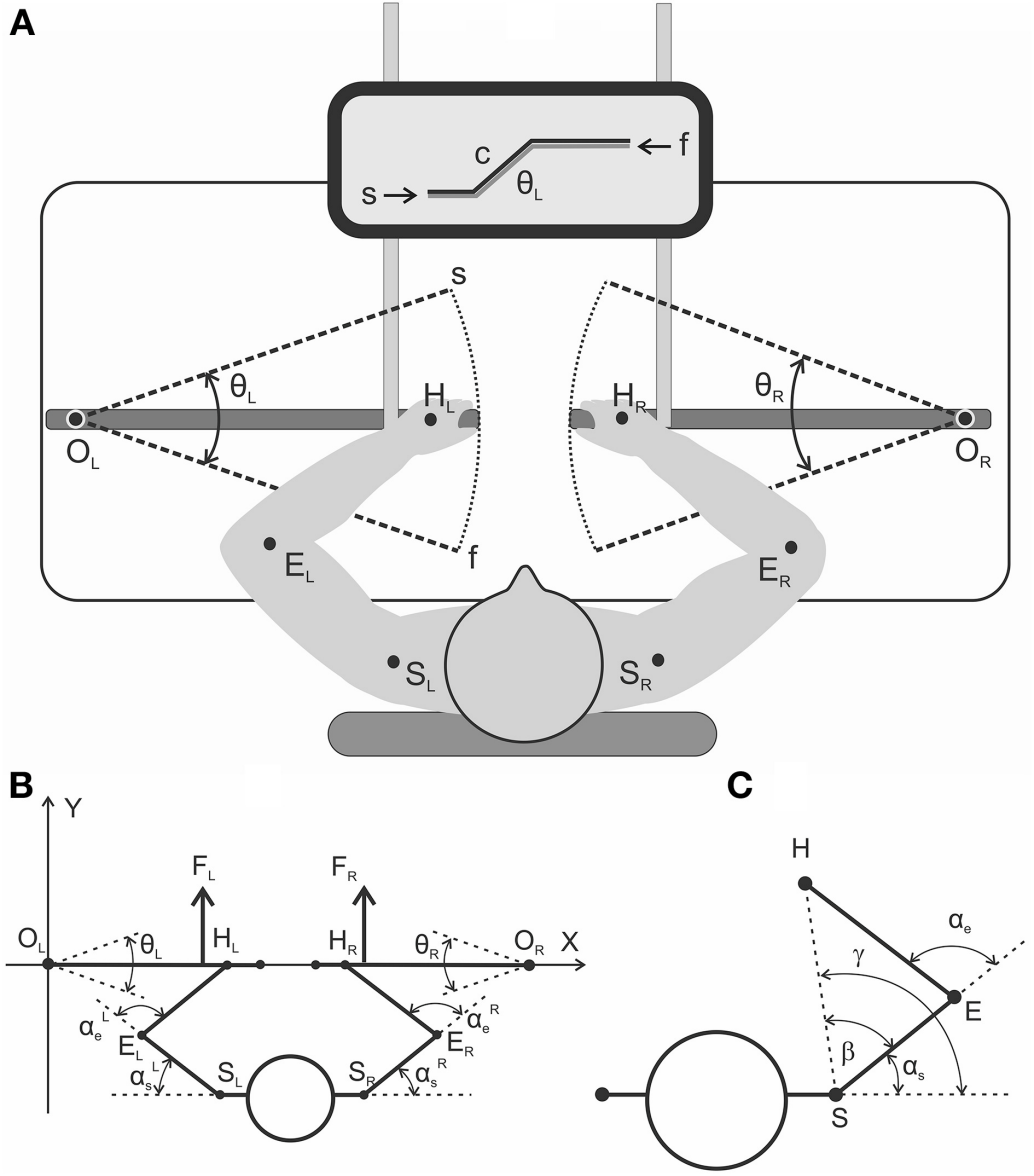
**(A)** general scheme of the experimental setup from a top view **(A)** and a detailed geometry description regarding the relative arrangement of the moving arm segments and levers **(B,C)**. **(A)** The subjects produced symmetrical bimanual movements using a visual tracking procedure. The target movements were executed via the combination of two beams on the monitor screen: the command signal (c) evolving with time as a trapezoid and the signal from the joint angle sensor of the left lever (θ_*L*_). **(B)** General scheme of the experimental setup from a top view with a detailed geometry description regarding the relative arrangement of the moving arm segments and levers. Real parameters of the arm's segments (*EH, ES*) and the distance between the rotation centers at the shoulders (*S*_*L*_*S*_*R*_) were defined for each subject prior to the experiment. **(C)** Geometrical drawing used to define the precise joint angle changes in the shoulder and elbow joints (α_*s*_, α_e_) based on real records of the levers' turning angles (θ_*L*_, θ_*R*_); a detailed description is provided in the text.

Experiments were conducted with nine adult right-handed men, 19–39 years old (24.8 ± 5.5). An informed consent was signed by each subject before the experiments. All study procedures were in accordance with the ethical standards of the institutional and/or national research committee of A. A. Bogomoletz Institute of Physiology, National Academy of Sciences, Kiev, Ukraine, and with the 1964 Helsinki declaration and its subsequent amendments or comparable ethical standards. Informed consent was obtained from all individual participants included in the study. The experimental procedure lasted ~1 h. The mechanical part of the experimental setup is schematically presented in Figure 1. The subject sat near a special table in a chair with a regulated position of the chair-bottom; his position was adjusted via the elevation of his armpits 10–15 cm over the table plate. The chair was rigidly fixed to the floor, and the subject's trunk was fixed to the chair back by special belts. The subject held two rotating wood levers that imitated boat oars; the levers could move around vertical axes (“oarlocks”) only in horizontal plane due to a special ball-bearing fixation to the axes. The plane of the levers' axes movement approximately coincided with positioning of the shoulder joints; therefore, it was most convenient for subjects to move the arms' segments in this plane without any additional suspension supports of his arms. Rubber bands (4 m length in the non-stretched state) connected the levers as shown in Figure 1A and created identical loads on the subject's arms; the loads could be increased by the connection of additional bands. In the initial positions of the levers prior to the beginning of movement (point *s* in Figure 1A), the subject's arms were fully extended; the initial forces that acted on the subject's hands (points *H*_*L*_, *H*_*R*_) were approximately 32 and 64 N for the single and double bands of loading properly. When the levers were pulled, the loads were raised linearly, which achieved the final positions (point *f* in Figure 1A) of 44 and 88 N. Precision potentiometers were used to measure the rotating angles of the levers (θ_*L*_; θ_*R*_); zero values of the signals were installed at the middle levers' positions as shown in Figure 1. The positive deflection corresponded to the pulling movement direction, which coincided with the clockwise turning of the left lever and the anti-clockwise turning of the right lever. The subjects executed the symmetrical bimanual pulling and returning movements through a combination on the monitor screen of the beam that reflected the angle position of the left lever (θ_*L*_) with the command signal (*c*) unwrapping over the screen as a trapezoid with a duration of the ramp phases 0.4, 1.0, or 2.0 s.

### Off-line computation of the joint angles

Figures 1B,C show definition of the movement parameters. A rigid mechanical coupling of the subject's arm segments with levers during movement enables a precise computation of the joint angle traces [α_e_(*t*), α_*s*_(*t*)] based on the change in position of the corresponding lever [turning angle, θ(*t*)]. In this scheme, the abscissa of the drawing passes through the centers of the levers' rotation *O*_*L*_ and *O*_*R*_, and the origin of the coordinates coincides with the left center (*O*_*L*_). The coordinates of the shoulder joints (points *S*_*L*_, *S*_*R*_) and the length of the shoulder and forearm segments (*SE* = *L*_*s*_; *EH* = *L*_*e*_) were defined prior to the initiation of the experiments; the positions of the hands at the levers were standard for all subjects (*R* = *OH* = 66 *cm*). Zero value of the turning angle (θ = 0) coincides with abscissa axis and positive values assumed for anti-clockwise rotation of the right lever and clockwise of the left one in a pulling direction. The coordinates of the hand positions (*H*_*L*_, *H*_*R*_) for the different turning angles of the levers are defined by the following trigonometry expressions:

(1) $$H_{R}=O_{R}+R\cdot \left[\begin{array}{c} -\cos\left(-\theta_{R}\right)\\ \sin\left(-\theta_{R}\right) \end{array}\right];H_{L}=O_{L}+R\cdot \left[\begin{array}{c} \text{cos}(-\theta_{L})\\ \sin(-\theta_{L}) \end{array}\right]$$

The formulae for the calculations of the angles at the shoulder (α_*s*_) and elbow (α_e_) joints are subsequently presented for the right arm; similar expressions are used for left arm. First, there are defined polar coordinates for the changing position of the hand at the plain:

(2) $$\begin{array}{l} L=\sqrt{{\left(H_{x}-S_{x}\right)}^{2}{+\left(H_{y}-S_{y}\right)}^{2}};\gamma =arctg\left(\frac{H_{y}-S_{y}}{H_{x}-S_{x}}\right), \end{array}$$

where *H*_*x*_, *H*_*y*_, *S*_*x*_, *S*_*y*_ are the corresponding Cartesian coordinates of the hand and shoulder joints, respectively.

Finally, the joint angles α_*s*_ and α_e_ are defined by the expressions (3) and (4):

(3) $$\begin{array}{l} \beta =arccos\left(\frac{L^{2}+L_{s}^{2}-L_{e}^{2}}{2\cdot L_{e}\cdot L_{s}}\right);\alpha_{s}=\gamma -\beta ; \end{array}$$

(4) $$\begin{array}{l} \alpha_{\text{e}}=\pi -\;arccos\left(\frac{L_{e}^{2}+L_{s}^{2}-L^{2}}{2\cdot L_{e}\cdot L_{s}}\right) \end{array}$$

### EMG recording and off-line handling

**Figure 2:**
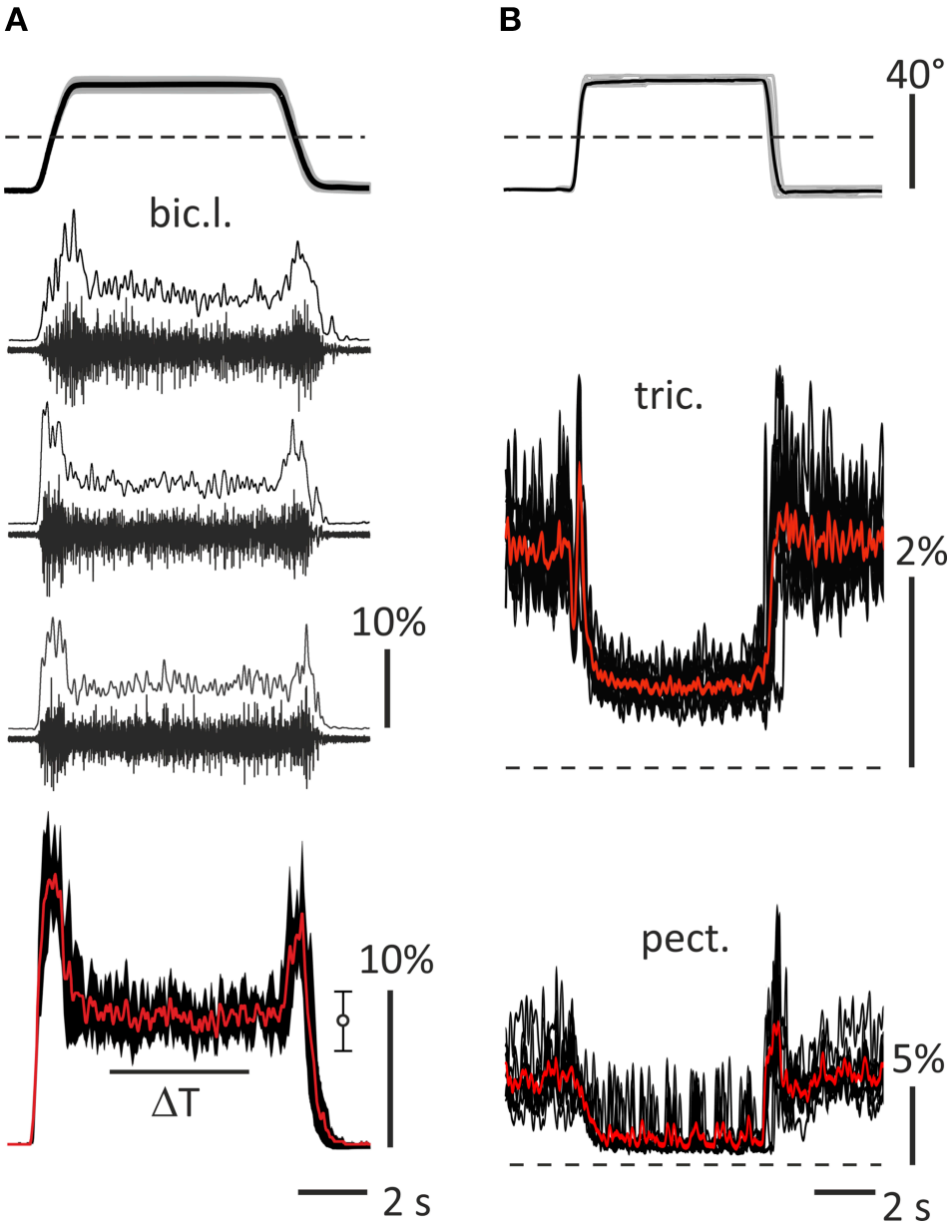
**General description of the data handling used in the study**. **(A)** A part of standard movement tests (subject 23A, left *bic.l*.); upper panel shows averaging of 10 records of the turning angle of the left lever (black) and their trial-to-trial variability (± *SD*, gray area), dash line marks zero angle corresponding to the middle lever′ positions in Figures 1A,B; middle panels (2–4)—single raw EMGs recorded in 10–5000 Hz range and results of the full wave rectification and smoothing by 4th order Batherworth low-pass filter with 10 Hz cut-off frequency; bottom panel—averaging of 10 smoothed records (red), three of which are shown in panels 2–4, and their trial-to-trial variability (± *SD*, black area); statistical parameters of the averaging within ΔT interval are presented at the right (*m* ± *SD*). **(B)** Examples of activity of the right *tric*. and *pect*. (subject 25B); upper panel show superposition of 10 turning angle records (gray) and their averaging (black); middle and bottom panels show superpositions of 10 smoothed rectified EMG records (black) and their averaging (red). The EMG calibration is given in % of MVC.

Surface EMGs were recorded using surface electrode pairs (Biopac System EL 503, USA; center to center distance 25 mm), which were fixed at both arms on the subject's skin over the muscles' bellies. The activity was registered from the following muscles: *pectoralis major, deltoideus scapularis, biceps brachii caput longum et breve, brachioradialis*, and *triceps brachii caput longum*. The recorded muscle activity was amplified via a multichannel amplifier (16-channel Bioamplifier, CWE, Inc., PA 19003 USA) using a bandpass filter in the range of 10–5000 Hz (Figure 2A). All raw EMG records were inspected visually, and possible mechanical artifacts (De Luca et al., 2010) were identified and removed. The EMGs together with the two position signals (θ_*L*_, θ_*R*_) were collected via a CED Power 1401 data acquisition system, using the program Spike 2 (Cambridge Electronic Design, UK). The EMGs and position signals were digitized at 10.0 and 2.0 kHz, respectively. Origin 8.0 (OriginLab Corporation, USA) and SPSS 17.0 (IBM Business Analytics software) were used for the off-line data analysis. Procedure of EMG recording and data handling is presented in Figure 2. The EMG records were full-wave rectified and additionally filtered (Batterworth filter of fourth order, bandwidth 0–10 Hz) in an off-line regimen; this procedure introduced a phase lag with respect to the real changes in the EMG intensity near 130–150 ms (Tal'nov et al., 2014). All tests were repeated 10 times to average the corresponding records. When necessary, the averaged trajectories of movement also underwent numerical differentiation to obtain the velocity and acceleration of the movement. Prior to each experiment, the maximal voluntary contractions (MVC) of all muscles were registered to normalize the averaged EMG records in the percentage of MVC. During the procedure, the maximal EMG intensities of the corresponding muscles were registered in either pulling or returning bimanual isometric contractions produced when a participant sat in a standard experimental position, and the elbow and shoulder joint angles constituted ~90° and 40°, respectively. Examples of the raw EMGs and various stages of the signals handling are presented in Figure 2 for *bic.l, tric*., and *pect*. muscles. Recording of the raw EMG in a single movement test and result of the low-pass filtering of the full-wave rectified records are shown for *bic.l*., rest of the EMGs include superpositions of 10 records and their averaging. The elbow flexors and shoulder extensors were almost inactive before and after test movements, therefore these parts of the records can be used to determine the zero activation levels. On the contrary, the elbow extensors and shoulder flexors usually demonstrated some activity before and after tests, the dashed lines in this and other Figures show additionally the zero levels of activity. The EMGs in *pect*. muscles in many cases are distorted by the ECG waves, and their averaged records contain clearly seen oscillations (Figure 2B, bottom panel). In the framework of the present experimental approach, we cannot completely exclude contamination of the EMG records by mechanical artifacts, which may be likely added during fast movements. The frequency range of these artifacts is not significantly different from that of the raw EMGs, therefore this noise cannot be removed by a simple filtering, and more sophisticated computation procedures must be applied.

### Estimation of the static and dynamic components in the averaged EMG records

**Figure 3:**
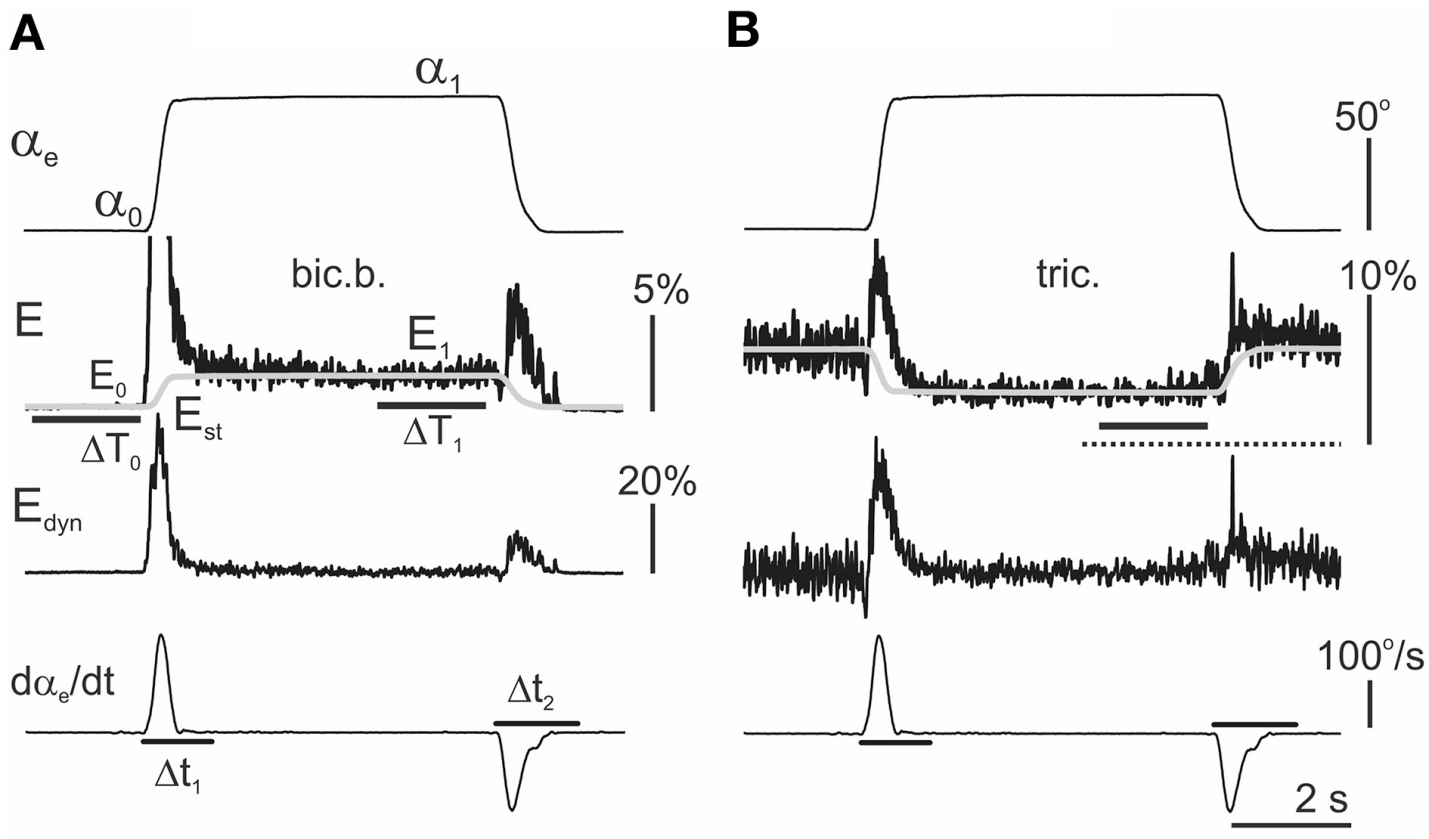
**Extraction of the dynamic components within the averaged EMG records (see detailed description in the text)**. The same procedure was applied to analyse the reactions in both the flexor **(A)** and extensor **(B)** muscles. First, the amplitude of the static component *E*_st_ was determined by averaging the EMG activities over 2 s periods Δ*T*_1_ prior to the end of the hold phases in the test movements. Finally, the time course of the static components in the EMGs was assumed to repeat the time course of the angle changes in the corresponding joint; Equation (5) had been proposed for its description (shown by the gray lines in the second row from top). The dynamic EMG components were determined using the difference between the initial EMG record and the static component evaluation *E*_st_. In flexors, the resulting dynamic components *E*_*dyn*_ = *E*−*E*_st_ predominantly include two different waves of activity connected directly with the phases of active muscle shortening (*D*_*P*_) or lengthening (*D*_*R*_). If the *E*_*dyn*_ traces are compared with the first derivative of the movement records (*d*α_e_*/dt*), one can conclude that the durations of *D*_*P*_ and *D*_*R*_ waves exceed the durations of the movement phases. Therefore, for the correct assessment of the areas of the components, the integration times were elongated 1.5 times with respect to the duration of the derivative waves (lines Δ*t*_1_ and Δ*t*_2_ in the bottom panels, *d*α_e_/dt graphs). The EMG calibration is given in % of MVC.

The following method was proposed to quantitatively compare the activation intensities of different muscles (Figure 3). This approach enables an evaluation of weights of the dynamic and static components in the averaged EMG records. First, within 2 s intervals Δ*T*_0_ and Δ*T*_1,_ two static levels were defined in the averaged EMG records: *E*_0_—the background EMG intensity, and *E*_1_—the stationary activation intensity prior to the start of the returning phase in the test movement (Figure 3A). *E*_0_ was primarily close to zero in the elbow flexors and shoulder extensors, and typically achieved small positive values in the elbow extensors and shoulder flexors. During the hold phase, the *E*_1_ increased compared with the *E*_0_ in the elbow flexors and shoulder extensors and primarily decreased in in the elbow extensors and shoulder flexors (Figure 3B). The static components *E*_st_ in the averaged EMG reactions were approximated using the following expressions:

(5) $$\begin{array}{l} E_{\text{st}}\left(t\right)=E_{0}+\frac{\alpha \left(t\right)-\alpha_{0}}{\alpha_{1}-\alpha_{0}}\left(E_{1}-E_{0}\right), \end{array}$$

where the parameters α_0_, *E*_0_, and α_1_, *E*_1_ represent the averaged values of the joint angle and EMG intensity computed within the 2 s intervals Δ*T*_0_ and Δ*T*_1_ prior to the movement phases.

The dynamic part of reaction was determined by subtraction from the initial EMG record the previously defined static component:

(6) $$\begin{array}{l} E_{dyn}=E-E_{st} \end{array}$$

The integral dynamic components (*I*_*dc*_), which describe the EMG activity changes during the movement phases, were defined by the integration of *E*_*dyn*_ within the correspondent time intervals Δ*t*_1_ and Δ*t*_2_. These intervals were evaluated using the first derivative of the correspondent joint angle changes (lowest rows in Figures 3A,B). Because the dynamic changes of the activity ordinarily exceeded the durations of the movement phases, the integration times Δ*t*_1_ and Δ*t*_2_ were subsequently obtained by elongation of the durations of the derivative waves 1.5 times. Finally, the integral dynamic components during the pulling (1) and returning (2) phases of the test movements were defined by the following expression:

(7) $$D_{P,\;R}=\frac{\int_{\Delta t_{1,2}}E_{dyn}(t)dt}{\Delta R},$$

where *E*_*dyn*_ is defined according to Equations (5) and (6); Δ*t*_1_ and Δ*t*_2_ represent the integration times during the pulling and returning phases, respectively; and Δ*R* represents the duration of the ramp phases in the command signal, i.e., 2.0, 1.0, or 0.4 s.

## Results

### The averaged EMGs recorded from the muscles of both arm

**Figure 4:**
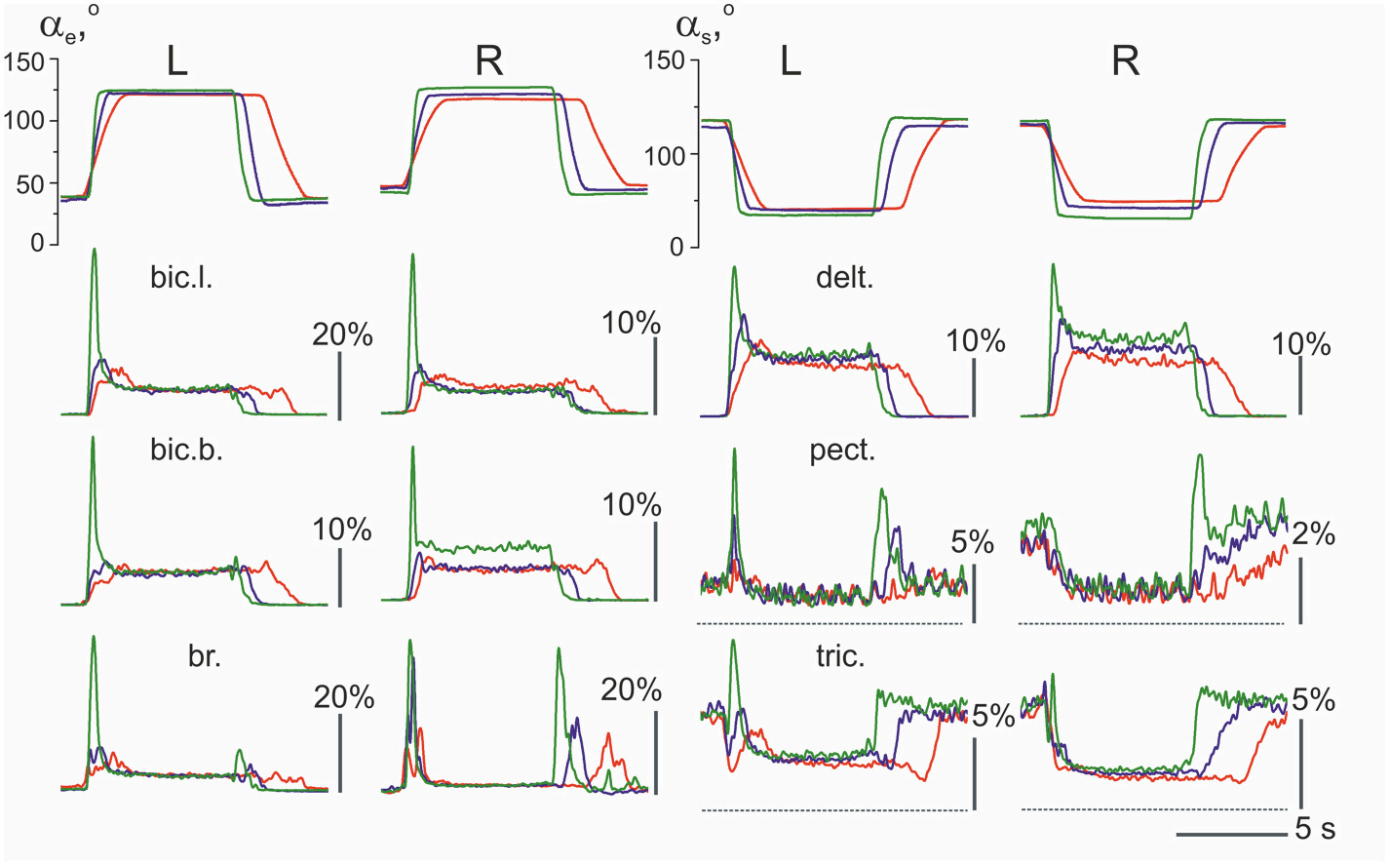
**The averaged joint angle trajectories and EMGs recorded from identical muscles in the *left* (L) and *right* (R) arms during test movements (*subject 25B*)**. Responses to the command signals with three durations of ramp phases 2.0, 1.0, and 0.4 s are compared; the corresponding reactions are marked by different colors. Two rubber bands were used at each side for loading the test movements; at the middle positions of the levers (as shown in Figure 1), the loads that acted in area of the subject's hands constituted approximately 76 N. EMG records are mainly placed below the angle traces of the corresponding joint, including the elbow (α_e_, left half of Figure) or shoulder (α_*s*_, right half); the triceps EMGs are shifted into the right half of the Figure to obtain a better picture format. Other abbreviations used in this and the following Figures: *bic.l., m. biceps brachia cap. longum*; *bic.b., m. biceps brachia cap. breve*; br., *m. brachioradialis*; delt., *m. deltoideus pars scapularis*; pect., *m. pectoralis pars major*; and tric., *m. triceps brachii caput longum*. Note that the *pect*. and *tric*. largely exhibited background activation at the initial position preceding the test movements; the dashed lines note zero levels of activity in the EMGs recorded from these muscles. Rest muscles were inactive before and after movement test, therefore the zero levels are presented by straight lines at the EMG records before and after movements. The EMG calibration is given in % of MVC.

An example of the averaged EMGs recorded from the same muscles in both arms is shown in Figure 4. The angle trajectory changes in the elbow (α_e_) and shoulder (α_*s*_) joints in both arms (*left*–L; *right*–R) were calculated based on θ_L_, θ_R_, which represent the turning angle records of the proper levers in conformity with the procedure described in Section Off-line Computation of the Joint Angles (Figure 1C). The dynamic components of the EMG reactions recorded during the pulling and returning phases of movement were quite different in various muscles and were dependent on the direction and velocity of the movement. In contrast, the steady-state activities during the hold phases were not noticeably different in their dependence on the velocity; however, these components occasionally varied, with a predominant tendency to increase at higher velocities of movement (see the EMG records from the right *bic.b*. and right *delt*. in Figure 4). In the test movements, the elbow flexor muscles (*bic.b., bic.l*., and *br*.) act in a similar way with the shoulder extensors (*delt*.); a certain similarity is also present in the reactions of the elbow extensors (*tric*.) and the shoulder flexors (*pect*.). The functional associations of the elbow flexors with the shoulder extensors and the elbow extensors with the shoulder flexors enable the selection of two groups of the synergist muscles that belong to different joints in antagonistic relationships with one another. The muscles of *PM group* (elbow flexors and shoulder extensors) generated powerful bursts of activity during the pulling movements; in contrast, their activities predominately decreased during the returning phases of the movement, when they contracted in the “yielding” regimen. In contrast to the muscles of *PM group, RM group* muscles (elbow extensors and shoulder flexors) typically exhibited a weak background activity in the initial position. The pulling movements in these muscles were associated with complex velocity-dependent oscillations of activity with a tendency to decrease; during the hold phases, the EMG intensities predominantly decreased until full disappearance. In the following returning phases of the movement, the EMG intensity in these muscles often recovered to a background level; thus, following an increase in the movement velocity, clear, dynamic oscillations of activity appeared.

When the EMG reactions in the same muscles that belong to different arms are compared, their similarities may often be noted, such as in the experiment presented in Figure 4. The subject who participated in this experiment demonstrated stable and similar EMG reactions in the same muscles in both arms. However, several differences exist, which consist, in particular, of velocity-dependent shifts in the stationary EMG levels in the *bic.b*. and *delt*. muscles in the right arm, whereas these shifts are absent in the left arm. These differences may be explained, at least in part, by the increased movement amplitudes produced by the right arm (compare the joint angle traces in Figure 4). The larger movement amplitudes of the right arm are likely connected with the absence of the direct visual control of its movement (see Methods). In contrast, the observed inconstancy of the EMG activities may also reflect the redistribution of activity among muscles of *PM group*, as well as a corresponding change in the opposing forces generated by *RM group*. In addition, it should be noted that the present experimental approach could not provide control of all muscles in the movement tests.

**Figure 5:**
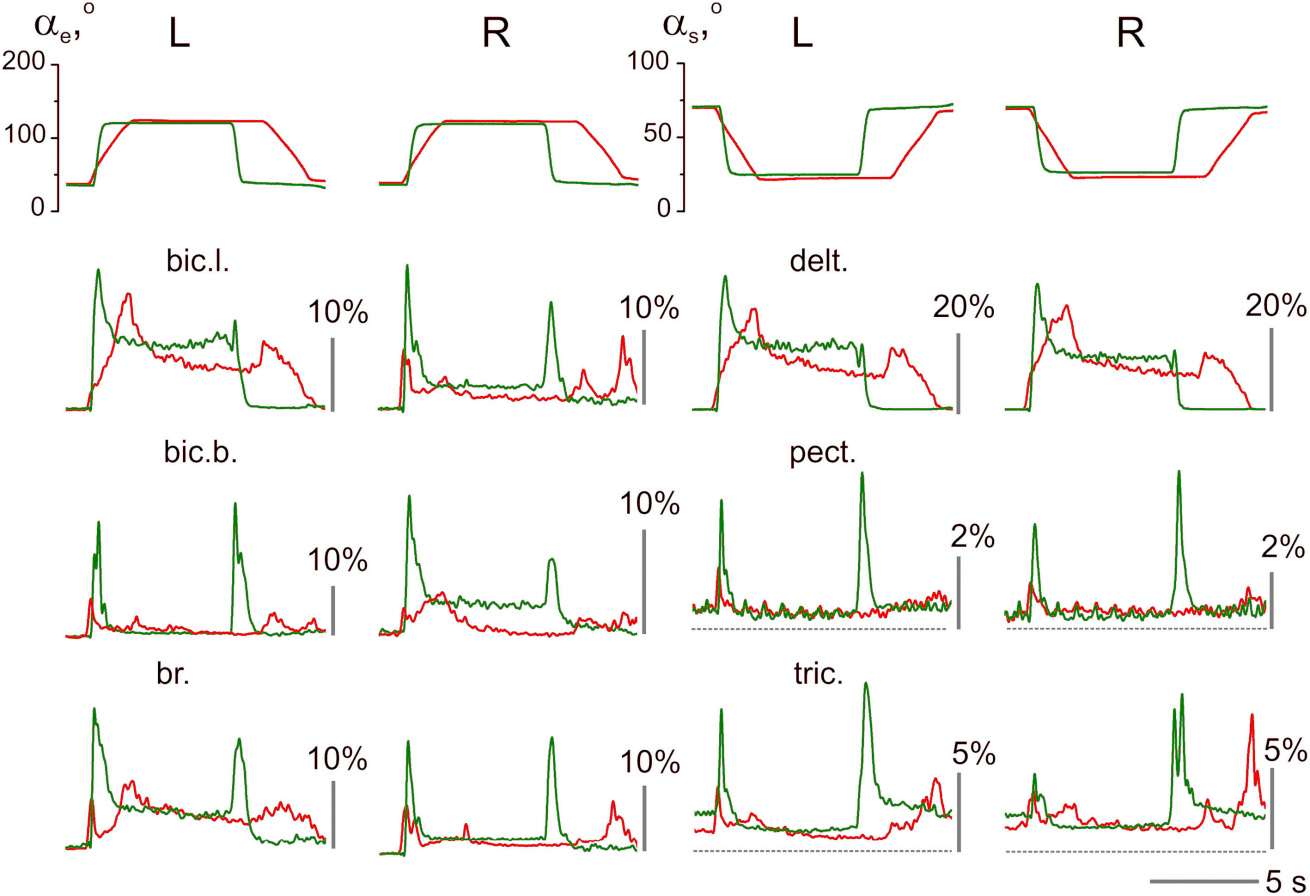
**Example of unstable patterns in the averaged EMG records; the test movements with 2.0 and 0.4 s ramp phases in the command signals are shown (*subject 25A*)**. The EMG calibration is given in % of MVC; the same designations as in Figure 4.

An example of the unstable EMG reactions with non-uniform distribution of activity among different muscles in both arms is presented in Figure 5. This subject exhibited quite good movement tracking and precise fixation of the hold positions by both arms; at the same time, in contrast with the experiment presented in Figure 4, the averaged EMG records were mainly different for the identical muscles that belonged to these arms. Within *PM group*, a similarity of reactions was registered only in the *delt*., whereas in all muscles of the elbow joint, the EMG reactions were remarkably different. A common peculiarity in the muscle reactions of *PM group* was evident dependent on the hold EMG levels on the movement velocity; an increase in the velocity typically led to an increase in the static EMG intensity. Another apparent property of these reactions was the presence of well-expressed dynamic components during the returning phases of the movements; moreover, in some muscles (left *bic.l*., left and right *delt*.), these components even exceeded the components recorded during slower movements. During fast movements, quite similar strong dynamic reactions appeared almost synchronously in *RM group* of muscles; therefore, the antagonist muscles that act at both joints were co-contracted in these cases. It should also be emphasized that these mainly unpredictable features of the central commands in this subject were not associated with a worse movement quality compared with the other subjects.

Velocity of movement is essential factor defining reactions of the elbow extensors and shoulder flexors, what is well seen in Figures 4, 5. At minimal velocity, subjects provide the necessary movement predominantly by lowering intensity of activation in these muscles. It seems that the drop of activity in these muscles become insufficient with velocity rise, what may be compensated for by the dynamic components of EMG in the elbow flexors and shoulder extensors, thus switching on a process of redistribution of the central commands between muscles-antagonists.

### Statistical analysis of the integral dynamic components of EMGs

**Figure 6:**
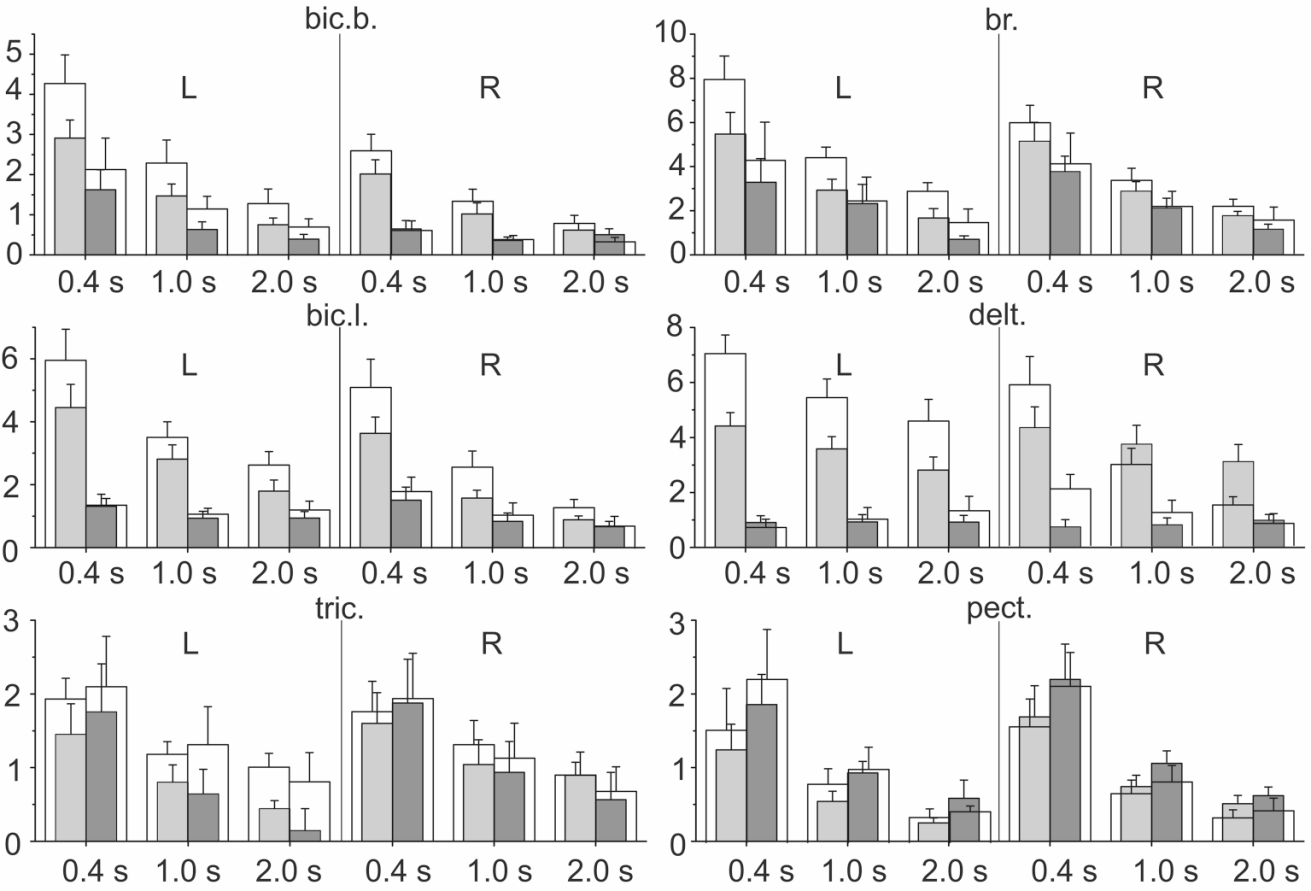
**Statistical analysis of the integral dynamic components of the EMGs during the pulling (*D*_*P*_) and returning (*D*_*R*_) phases of the test movements produced with three different movement durations and two values of external load (*m* ± *SE*, group of nine subjects)**. For each EMG recording at a given ramp duration, four parameters are shown: the *gray (white) bars* correspond to the tests for one (two) rubber band loading; the left pairs of bars (*light gray and white*) describe *D*_*P*_, and the right pairs (*dark gray and white*) describe the *D*_*R*_ components. The numbers under the separate bar groups (abscissa axes) signify the duration of the ramp phases; *L, R* indicate left and right arms, respectively; ordinates are given as the percentage of MVC.

The integral dynamic components *D*_*P*_ and *D*_*R*_ (in accordance with the quantitative method described in Section Estimation of the Static and Dynamic Components in the Averaged EMG Records) are summarized in Figure 6 for the group of nine subjects; these parameters have been defined at three durations of the test movements and two levels of loading. A Four-way ANOVA for repeated measurements was applied to estimate the potential dependences of the dynamic components on the used experimental conditions for each particular muscle (Table 1). The following independent factors are taken into account: **D**—the movement direction factor: pulling (*D*_*P*_) or returning (*D*_*R*_); **S**—the side factor: the *left* (L) or *right* (R) arm; **P**—the load factor: one or two rubber bands; and **V**—the movement velocity factor (ramp duration: 0.4, 1.0, or 2.0 s). In *PM group* of synergic muscles (*bic.b., bic.l., br., delt*.), in most cases, the *D*_*P*_ components were significantly increased compared with the *D*_*R*_ components; however, at lower movement velocities (1.0, 2.0 s), these differences were less expressed. Moreover, in some cases, a reverse ratio between the component values was identified. Nevertheless, while considering the influence of the velocity factor on the dynamic components of EMG, significant effects were identified in all studied muscles (Table 1). The action of the load factor was significant in all muscles of *PM group*, whereas the muscles of *RM group* did not react in this way, which could, at least in part, be connected with the low intensity and instability of their reactions (***P*** column in Table 1). A significant action of the side factor was fixed only in the *bic.b*. reactions (***S*** column in Table 1). Significant differences in the *D*_*P*_ component at different sides were identified for the double loading and all durations of movement (0.4, 1.0, and 2.0 s); the *D*_*R*_ component exhibited similar differences for the single band loading and 0.4 s of movement duration (Bonferroni *post-hoc* analysis *p* < 0.05).

**Table 1:** **Four-way ANOVA with repeated measurements for the dynamic EMG components**.

	**D**	**S**	**P**	**V**	**DS**	**DP**	**SP**	**DSP**	**DV**	**SV**	**DSV**	**PV**	**DPV**	**SPV**	**DSPV**
*bic. b*.	0.019	0.037	0.000	0.001	0.720	0.071	0.034	0.882	0.023	0.083	0.874	0.048	0.273	0.753	0.679
*bic. l*.	0.000	0.129	0.001	0.000	0.183	0.005	0.938	0.871	0.003	0.704	0.375	0.413	0.034	0.074	0.055
*br*.	0.015	0.685	0.034	0.016	0.517	0.227	0.211	0.226	0.089	0.727	0.299	0.164	0.010	0.392	0.153
*tric*.	0.882	0.789	0.746	0.003	0.760	0.923	0.178	0.685	0.209	0.740	0.197	0.500	0.775	0.316	0.164
*delt*.	0.000	0.330	0.002	0.007	0.897	0.000	0.024	0.152	0.000	0.862	0.376	0.004	0.000	0.101	0.915
*pect*.	0.008	0.788	0.930	0.012	0.582	0.645	0.392	0.567	0.166	0.516	0.878	0.712	0.422	0.669	0.369

*The following parameters are considered:* **D**—*direction factor (D_P_–pulling/D_R_–returning);* **S**—*side factor (left/right arm);* **P**—*load factor (one/two rubber bands);* **V**—*factor of the movement velocity (ramp duration: 0.4, 1.0, or 2.0 s). The header of table consists of marks of factors and their interactions. Combination of letters in header marks the interaction of respective factors. The cells with p < 0.05 are marked by gray color*.

The combined actions of various factors (**DP**, **SP**, **DV**, **PV**, **DPV**) may provide additional information regarding the central programs in the bimanual movements (Table 1). Weakness and frequent instability in the reactions of *the RM group* muscles are accompanied by the absence of a significant interaction of the factors (see *tric*. and *pect*. rows in Table 1). In contrast, this type of interaction was identified in the *delt*. muscle, which demonstrated highly repeatable, powerful and stable reactions in all experiments. Most likely, because of this stability, all combinations of the factors exerted a significant influence on the dynamic components of the EMGs in this muscle; however, these combinations were only partly effective in the biceps and completely ineffective in the *br*. During the movement phases, the central commands to the elbow flexors were more flexible and variable compared with the *delt*.; however, the *br*. could be identified in this muscle group because of a relative weakness of the steady-state reactions and, in many cases, the somewhat higher amplitudes of the *D*_*R*_ components (Figure 4). Peculiarities in the EMG reactions in the *br*. were also characterized by a significant combined action of three factors (***DPV***); however, the same combination of the factors was significant in both the *delt*. and *bic.l*. (Table 1).

### Statistical analysis of relative differences between the integral dynamic components at the pulling and returning movement phases

Based on the pattern of the integral dynamic components in *PM group* of synergic muscles (two upper rows in Figure 6), one can assume that *D*_*R*_ components are likely less expressed in the *delt*. muscle compared with the elbow flexors. To further confirm this assumption, we introduced the difference coefficients that define the relative differences between the integral dynamic components that belong to the pulling and returning movement phases:

(8) $$\begin{array}{l} k=\frac{\left({\bar{D}}_{P}-{\bar{D}}_{R}\right)}{\left({\bar{D}}_{P}+{\bar{D}}_{R}\right)∕2}, \end{array}$$

where ${\bar{D}}_{P}$ and ${\bar{D}}_{R}$ indicate the mean values of *PM* and *RM* integral dynamic components in the group of nine subjects (i.e., the bar amplitudes in Figure 6).

**Figure 7:**
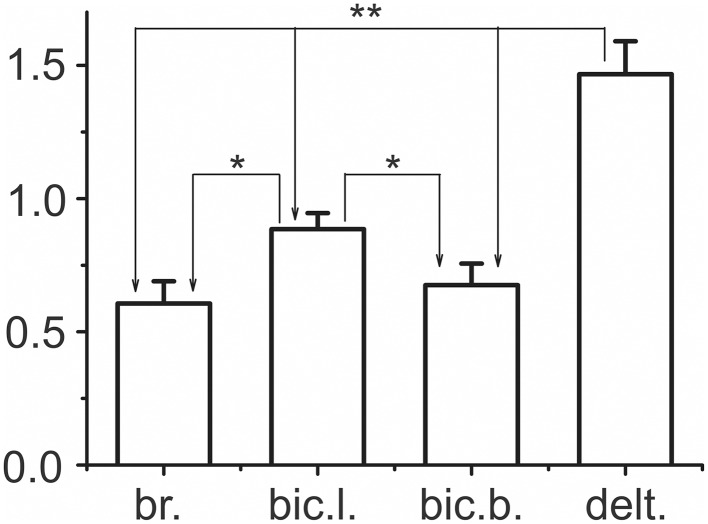
**Statistical analysis of the difference coefficients determined by Equation 8**. The difference coefficients were defined for the mean values *D*_*P*_ and *D*_*R*_ presented as the bar amplitudes in Figure 6. Shared populations of the coefficients for each of the four muscles, *bic.b., bic.b. br*., and *delt*., included the results obtained for all movement velocities at both arms, which thus consisted of 12 values for each muscle under study. The potential differences in the coefficients were analyzed by One-way ANOVA with repeated measurements, which indicated their significant dependence within a given group of synergist muscles (*F* = 309.788, *p* < 0.001). The results of the Bonferroni *post-hoc* analysis of pairwise comparisons for particular muscles are schematically indicated by the arrowed lines; one and two asterisks above the lines signify *p* < 0.05 and *p* < 0.005, respectively. The EMG calibration is given in % of MVC.

In accordance with this previous definition of the difference coefficients, maximum or minimum values (+2 or −2, respectively) will be achieved in the following conditions: 1) ${\bar{D}}_{P}\neq 0;\;{\bar{D}}_{R}=0$ and 2) ${\bar{D}}_{P}=0;\;{\bar{D}}_{R}\neq 0$; zero values of the coefficient will correspond to the condition:${\bar{\;D}}_{P}={\bar{D}}_{R}\neq 0$; its positivity (negativity) would signify that ${\bar{D}}_{P}({\bar{D}}_{R})$ prevails. When various muscles of the group (*bic.b., bic.b., br*., and *delt*.) were compared with respect to the difference coefficients, the distinctions between the arms, movement velocities, and loading levels were not taken into account; therefore, the coefficient sets included 12 quantities for each muscle. The sets of the difference coefficients were analyzed using One-way ANOVA with repeated measurements for each specific muscle; significant differences in a parameter were identified within *PM group* (*F* = 309.788, *p* < 0.001). The statistical characteristics of the difference coefficients are shown in Figure 7; the results of the Bonferroni *post-hoc* analysis of the pairwise comparisons for specific muscles are schematically presented by the corresponding arrowed lines. The positivity of the coefficients for all muscles of the group signifies that the first dynamic coefficient is higher compared with the second value. Therefore, it could be concluded that weights of the second dynamic components were smaller in the shoulder extensor muscle (*delt*.) compared with the elbow flexors.

### Statistical analysis of the static components of the EMGs in the test movements

**Figure 8:**
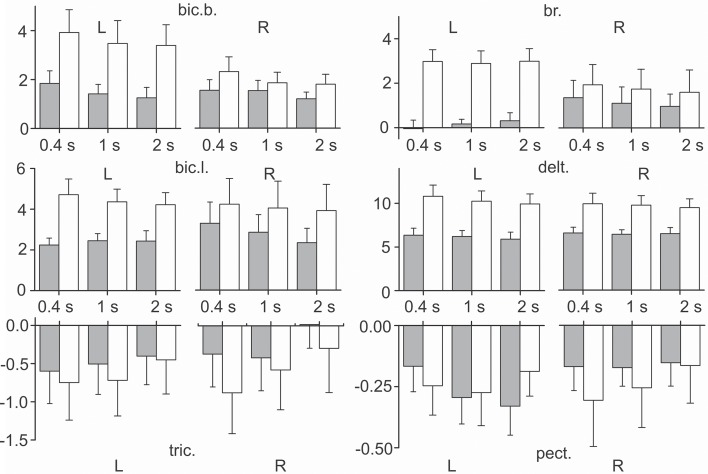
**Statistical analysis of the static components of the EMGs in the test movements produced with three different movement durations and two values of external load (*m* ± *SE*, *N* = 9)**. Tests with loading by one and two rubber bands are shown as *gray* and *white bars, respectively*; the other designations are similar to Figure 6. Note that the negativity in the reactions of the *mm*. *triceps, pectoralis* indicate that the levels of their stationary activity decrease with respect to their initial states. The EMG calibration is given in % of MVC.

A Three-way ANOVA for repeated measurements for all muscles was used to estimate the dependence of the static component *E*_st_ (Equation 5) on the **S**, **P**, and **V** factors. The muscles of *RM group* did not exhibit dependency on any of these factors, whereas all muscles of *PM group* depended on **P** factor; the static component increased with an increase in the external load (Figure 8). In addition, it was observed that the static component of the *bic.b*. significantly increased in the left arm compared with the right arm for the higher loads (two rubber bands) and slower movements (1.0 and 2.0 s ramp durations). An ANOVA analysis indicated a significant action of the interaction between the ***S*** and ***P*** factors in the reactions of the *bic.b*. and *br*.; a *post-hoc* Bonferroni analysis supported the existence of significant differences in these cases (*p* < 0.05). Noticeable dynamic components in the reactions of the *br*. muscle were associated with a relative weakness and instability of the stationary components, especially with a small loading. For the one band load, the mean values of the parameter in the left arm registered quite low; higher stationary values of the EMG activities in the right *br*. were simultaneously present with the high dispersion levels (Figure 8).

## Discussion

The present study was devoted to the analysis of simple, visually tracked, bimanual, symmetrical, ramp-and-hold movements executed against similar elastic loads. The focus of the investigation was to identify the patterns of the averaged EMGs in different muscles that act at the elbow and shoulder joints and to compare the reactions of similar muscles in the left and right arms. We did not take into account the hand muscle activity, which evidently plays an important role in the test movements; for a sake of simplicity, a complex multi-joint movement was reduced to the two-joint movement. The bimanual two-joint movements were fulfiled in the horizontal plane; these movements included symmetrical pulling and returning phases separated by the position fixation. Despite the simplicity, these movements are provided by the concerted action of many muscles; therefore, recording surface EMGs only in part of the muscles can present rather restricted information regarding the central commands that provide these movements. Moreover, the reliability of the EMG measures in the characterisation of activity patterns is known to be dependent on the variability that can occur in electrode placements. The proximity of the EMG electrodes to a muscle's innervation zone can affect the EMG signal (De Luca and Contessa, 2012), and the innervation zone may exhibit considerable variability in its location for certain muscles (Rainoldi et al., 2004). We understand that the influence of these problems become more essential if the EMG activities in similar muscles of different limbs are compared, which was accomplished in the present study.

Velocity of movement is essential factor defining reactions of the elbow extensors and shoulder flexors, what is can be seen in Figures 6, 7. At minimal velocity, subjects provide the necessary movement predominantly by lowering intensity of activation in these muscles. On the other hand, the drop of activity in these muscles become insufficient with velocity rise, what may be compensated for by the dynamic components of EMG in the elbow flexors and shoulder extensors. The simultaneous appearance of intensive dynamic EMG components in muscles-antagonists may reflect a tuning process of redistribution of activity between these muscles during fulfillment of fast movements.

*PM group* of synergy consists of the muscles that flex the elbow joint and extend the shoulder joint; these muscles provide an active pulling movement at the beginning of the test, support steady-state positions during the hold phase, and work in the yielding regimen during the returning phase. The general forms of the EMG intensity changes in actively contracting muscles and their rearrangement with an increase in the movement velocity are well corresponded with the reactions of the elbow flexors in single-joint isotorque movements (Kostyukov and Tal'nov, 1991). The central commands to the muscles during ramp-and-hold flexing movements also include the dynamic and static components, which are crucially dependent on the muscle hysteresis (Kostyukov and Korchak, 1998); it has been assumed that hysteresis effects allow the intensity of the coming efferent activity to be diminished for clamping the muscle length after shortening (Kostyukov, 1998). During the returning phase of the test movements, when the muscles lengthen in the regimen of the yielding work, more complex reactions are registered (Figures 4, 5). Fundamental difficulties in the explanation of muscle behavior during lengthening exist even for experiments on nerve-muscle preparations in which the intensity in the incoming efferent activity can be completely controlled (Kostyukov, 1987, 1998). For the analysis of a real multi-joint movement, these difficulties are raised because of the restricted quantity of the muscles that produce the EMG records; another problem consists of the potential redistribution of activity among muscles, which is likely needed in the development of additional methodical approaches with a higher time resolution compared with the averaging technique.

*RM group* of synergy includes the muscles that are in antagonistic relationships with the muscles of *PM group*. During both movement phases, these muscles predominantly increase their activities, which thus opposes the forces generated by the actively contracting muscles of *PM group*. At the same time, during the hold phases of the test movements, they largely decrease activity, which therefore assists the actions of these muscles. The relatively low amplitudes of the dynamic components and frequently observed variability in the activity during the hold phases in these muscles are likely connected with their predominately subsidiary role in the given movements. It should be noted that the division of the muscles under study in accordance with their attitude to a definite joint is oversimplified. The places of the force applications are single-valued only for mono-articular muscles, such as the *bic.l., br*., and *delt. pect*., whereas the *bic.b. (m. biceps brachii breve)* and *tric. (m. triceps brachii caput longum)* are bi-articular muscles; however, these bi-articular muscles primarily provide movements around the elbow joint (van Bolhuis et al., 1998).

A common feature of *PM* and *RM groups* of synergic muscles consists of the strong dependency of the dynamic components on movement velocity, whereas the actions of the other factors, as well as the combinations of different factors are significant only in the muscles of *PM group* (Tables 1, 2). Side-dependent differences are identified only for the dynamic components of the EMG reactions in the *bic. b*.; in the other muscles, these differences are not significant. In all muscles of *PM group*, the load factor influences the steady-state EMG reactions, whereas the side and velocity factors do not evoke effects (Table 2). The strong action of the load factor is likely the main reason that the combination of the load and side factors is also effective in the *br*. and *bic. b*.

**Table 2:** **Result of the Three-way ANOVA with repeated measurements for static components**.

	**S**	**P**	**V**	**SP**	**SV**	**PV**	**SPV**
*bic. b*.	0.081	0.005	0.151	0.010	0.378	0.451	0.529
*bic. l*.	0.977	0.000	0.125	0.266	0.500	0.465	0.187
*br*.	0.912	0.002	0.695	0.004	0.260	0.765	0.149
*tric*.	0.544	0.386	0.053	0.405	0.092	0.613	0.127
*delt*.	0.756	0.000	0.290	0.093	0.515	0.870	0.163
*pect*.	0.446	0.844	0.057	0.423	0.054	0.053	0.530

*Similar symbols as in Table 1*.

A set of the efferent activities that control the two-joint test movements can be localized within separate time zones, in which the programs of co-contraction (co-activation) or reciprocal activation predominate. The movement phases are primarily accompanied by antagonist co-contractions, whereas the steady-states are connected with a preferential use of reciprocal activation. Recently, the movement dynamic under these basic patterns of the antagonist activations was studied via the experimental model of two antagonistic muscles (Gorkovenko et al., 2012). It has been demonstrated that a reciprocal activation pattern can essentially linearise the movements after a change in their direction, providing they also exhibit a fast beginning; the co-contraction patterns can distinctly reduce the undesirable hysteresis after-effects, such as the ongoing residual movements at the apexes of activity. Thus, the co-activation of the antagonistic muscles could reduce the uncertainty effects in the motor control system connected, in particular, with muscle hysteresis (Kostyukov, 1998; Gorkovenko et al., 2012). Behavioral studies of postural tasks have demonstrated that subjects use muscle co-contraction as a strategy to stabilize limb joints in the presence of external loads (Kearney and Hunter, 1990; De Serres and Milner, 1991; Milner and Cloutier, 1998); these subjects are also able to independently modulate the relative balance of the co-contraction and limb stiffness in different spatial directions (Gomi and Osu, 1998; Burdet et al., 2001) and at different joints (Gribble and Ostry, 1998). It has been suggested that the CNS may use co-contraction as a strategy to facilitate the accuracy of the limb movement (Gribble and Ostry, 1998; Gribble et al., 2003).

Despite a sufficiently good quantity of execution of the test movements by all subjects in the present study, the patterns of the EMG reactions are essentially diverse. In some subjects, the reactions of the identical muscles that belonged to the left and right arms were quite similar. Simultaneously, the activities recorded during the hold phases in these subjects did not contain noticeable oscillations or substantial trends; thus, the mean EMG intensities in these phases were close to one another for the different velocities of movements executed at the same levels of loading (Figure 4). In contrast, in other subjects, essential and often unpredictable side- or velocity dependent changes in the basic EMG components were identified (Figure 5). Our data indicate that the EMG variability during the hold sections of movements can differ in the muscles of *PM group*. The quality of the test executions was almost identical in all subjects; therefore, the variability of the EMG reactions may signify the presence of the activation redistribution among different muscles or motor units within the same muscles. One can speculate that the activity redistribution processes could decrease the development of fatigue in the actively contracting muscle groups, which has been demonstrated for the natural labor movements of professional butchers (Madeleine et al., 2003). However, muscle fatigue itself can essentially modify the central programs of movement execution (Lacquaniti, 1992; Haruno and Wolpert, 2005; Huffenus et al., 2006; Prilutsky et al., 2009; Fuller et al., 2013; Lampropoulou and Nowicky, 2014; Rampichini et al., 2014); thus, the observed EMG rearrangements might be, at least in part, secondary with respect to the fatigue effects. Nevertheless, we can assume that different subjects use different motor strategies, which differ to various extents, of the activity rearrangement among different muscles or within these muscles. In our opinion, the analysis of these processes might be expanded by the simultaneous recording of activities from different motor units; however, there are known methodological difficulties in using this approach during large-scaled movements under noticeable loads (Tal'nov et al., 2014). Thus, indirect methods of recording appear to be more valid for these purposes (Akazawa and Okuno, 2000). The identification of potential side-dependent asymmetries in the central motor programs cannot likely be effective using standard EMG methods, such as in the present study; however, some prerequisites for the existence of this asymmetry in spinal cord circuitries have recently been demonstrated in animal experiments (Pilyavskii et al., 2013). In the present study, the right-handed subjects participated, and experiments were not directed on searching possible asymmetries in the EMG patterns related to the handedness. Nevertheless, we suppose that application of the present experimental approach to more complex movement paradigms, which are traditionally elaborated in classical studies of bimanual coordination (Walter and Swinnen, 1992; Swinnen et al., 1995), might be suitable for analysis of more intimate processes within the motor control system.

## Conclusions

The muscles of *PM group* (which flex the elbow joint and extend the shoulder joint) generated noticeable, velocity-dependent, dynamic EMG components during the pulling and returning phases of the trapezoidal test movements, which supports a steady-state activity during their hold phases. The muscles of *RM group* (which extend the elbow joint and flex the shoulder joint) co-contracted with *PM group* during the movement phases and decreased activity during the hold phase. A Multi-way ANOVA analysis for particular muscles demonstrated that in both muscle groups, the dynamic components of the EMGs strongly depended on the velocity factor, whereas the side and load factors, as well as the combinations of various factors, were significant only in the muscles of *PM group*. The extent of the EMG variability changed in various subjects, which could signify that the same movements may be realized by central commands with different extents of activity redistribution among muscles. It has been assumed that the activity redistributions may decrease the development of fatigue effects in actively contracted muscles.

## Author contributions

TT, data analysis and discussion; AG, statistical analysis and data discussion; AT, data analysis and discussion; TA, data analysis and discussion; VM, data discussion; IV, data analysis and discussion; AK, data analysis and discussion.

### Conflict of interest statement

The authors declare that the research was conducted in the absence of any commercial or financial relationships that could be construed as a potential conflict of interest.

## Abbreviations

bic.b.: m. biceps brachii caput breve
bic.l.: m. biceps brachii caput longum
br.: m. brachioradialis
delt.: m. deltoideus pars scapularis
EMG: electromyogram
MVC: maximal voluntary contraction
pect.: m. pectoralis pars major
tric.: m. triceps brachii caput longum
PM: “pulling” muscles
RM: “returning” muscles.
